# Klaus Unsicker: in honor of his eightieth birthday

**DOI:** 10.1007/s00441-021-03566-w

**Published:** 2022-01-03

**Authors:** Horst-Werner Korf

**Affiliations:** Zentrum Anatomie Und Hirnforschung, Institut f. Anatomie 1, Universitätsstr. 1, 40225 Duesseldorf, Germany


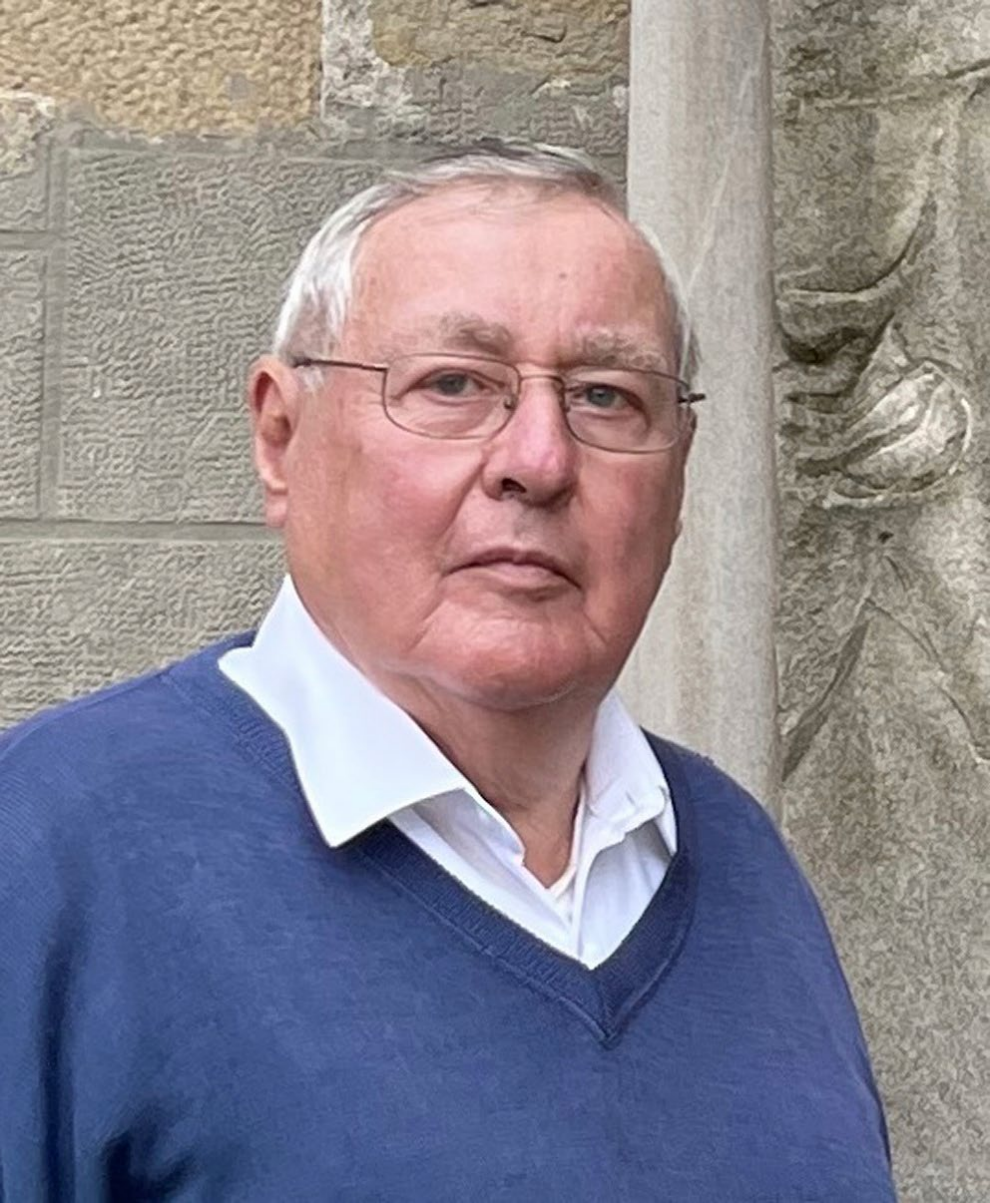
If you interact with Klaus Unsicker, you would not believe that he turns 80 on January 3, 2022. On this occasion, the editors and editorial office of Cell and Tissue Research, Springer Nature, and his numerous friends and colleagues all over the world would like to congratulate Klaus Unsicker and wish him health, productive activity, and vitality for many years to come. While former editorials (Franke 2012; Korf 2012) have paid tribute to Klaus Unsicker’s accomplishments as professor, researcher, and academic ambassador, this editorial will focus on his impact on the journal Cell and Tissue Research (CTR). Succeeding Andreas Oksche, Klaus Unsicker became coordinating editor in 1996 and ever since he has striven to improve the quality, the visibility and last but not least the impact factor of the journal. The introduction of special issues with focus on timely research projects was one of many innovations implemented during Professor Unsicker’s tenure. This initiative has become a great success, and till now, 46 special issues have been published (see Table 1), and the following statements reflect the role of Klaus Unsicker as stimulating leader of the editorial board:

**Table 1:** Cell and tissue research: special issues list

Title, volume	Guest editors	Editor	Papers	Pages
**Glial cell line-derived neurotrophic factor** (Vol. 286, No. 2, 1996)	K. Unsicker	K. Unsicker	12	104
**Molecular bases of axonal growth and pathfindings** (Vol. 290, No. 2, 1997)	U. Drescher, A. Faissner R. Klein, FG. Rathjen, C. Stürmer	K. Unsicker	34	285
**Molecular bases of limb and muscle development** (Vol. 296, No. 1, 1999)	R. Zeller	K. Unsicker	22	219
**Apoptosis 2000** (Vol. 301, No. 1, 2000)	J. Reed, M. Weller	K. Unsicker	15	204
**Recent advances in developmental neuroscience** (Vol. 305, No. 2, 2001)	K. Unsicker	K. Unsicker	12	115
**The circadian system: circuits–cells–clock genes** (Vol. 309, No. 1, 2002)	JH. Stehle	H. W. Korf	18	199
**Vasculogenesis and angiogenesis** (Vol. 314, No. 1, 2003)	R. Adams	K. Unsicker	18	177
**The dopaminergic nigrostriatal system: development, physiology, disease** (Vol. 318, No. 1, 2004)	O. von Bohlen und Halbach, K. Krieglstein, A. Schober, JB. Schulz	K. Unsicker	26	288
**Reproduction, development, and the early origins of adult disease** (Vol. 322, No. 1, 2005)	AE. Drummond, M. Wlodek	G. Risbridger	21	181
**The synapse – recent advances** (Vol. 326, No. 2, 2006)	M. Frotscher, E. Gundelfinger P. Jonas, E. Neher, P. Seeburg	K. Unsicker	34	468
**Stem cells: established facts, open issues, and future directions** (Vol. 331, No. 1, 2008)	G. Kuhn, O. Brüstle, U. Martens, A. Wobus	K. Unsicker	28	372
**Endothelial cell biology and pathology** (Vol. 335, No. 1, 2009)	E. Dejana, H. Wolburg, M. Simionescu	W. W. Franke	20	300
**Cell interactions with the extracellular matrix** (Vol. 339, No. 1, 2010)	L. Bruckner-Tuderman, K. Von Der Mark	T. Pihlajaniemi	21	280
**Innate immunity** (Vol. 343, No. 1, 2011)	B. Singh, G. Mutwiri, P. Griebel	B. Singh	21	261
**TGF-ß in aging and disease** (Vol. 347, No. 1, 2012)	K. Krieglstein, K. Miyazono, P. ten Dijke	K. Unsicker	27	301
**Endogenous musculoskeletal tissue regeneration** (Vol. 347. No. 3, 2012)	D.W. Hutmacher, G. Duda, R.E. Guldberg	K. Unsicker	27	345
**Molecular Biology meets Cardiology (Special Workshop “Heidelberg Heart II”)** (Vol. 348, No. 2, 2012)	W.W. Franke, W. Birchmeier	W.W. Franke	10	121
**Molecular bases of neural repair mechanisms** (Vol. 349, No. 1, 2012)	H.W. Müller, M. Sendtner, M. Bähr	K. Unsicker	30	404
**Cell biology solves mysteries of reproduction** (Vol. 349, No. 3, 2012)	P. Sutovsky	P. Sutovsky	19	264
**Current insights into protease dynamics in human epithelial disease and barrier function** (Vol. 351, No. 2, 2013)	M.A. Curtis, D.P. Kelsell	D.P. Kelsell	12	139
**Cell-to-cell communication: current views and future perspectives** (Vol. 352, No. 1, 2013)	H.–H. Gerdes, R. Pepperkok	K. Unsicker	13	177
**Neuroprotection in Glaucoma** (Vol. 353, No. 2, 2013)	E.R. Tamm, F. Grehn, N. Pfeiffer	K. Unsicker	15	153
**Rodent models of psychiatric disorders – practical considerations** (Vol. 354, No. 1, 2013)	P. Gass, C. Wotjak	K. Unsicker	24	330
**Between sealing and leakiness: molecular dynamics of the endothelium to maintain and regulate barrier function** (Vol. 355, No. 3, 2014)	H. Schnittler	K. Unsicker	20	256
**Epigenetics: Development, Dynamics and Disease** (Vol. 356, No. 3, 2014)	T. Vogel, S. Lassmann	K. Unsicker	18	213
**Dysfunction of neuronal calcium signaling in aging and disease** (Vol. 357, No. 2, 2014)	A.M.M. Oliveira, H.Bading, D. Mauceri	K. Unsicker	10	122
**Deciphering the core instructions of neuronal differentiation** (Vol. 359, No. 1, 2015)	U. Ernsberger	K. Unsicker	25	384
**Quantitative Techniques for Imaging Cells and Tissues** (Vol. 360, No. 1, 2015)	C. von Bartheld, F. Wouters	K. Unsicker	14	194
**Junctions in human health and inherited disease** (Vol. 360, No. 3, 2015)	S. Getsios, D. P. Kelsell, A. Forge	D. P. Kelsell	25	348
**Auditory system: development, genetics, function, aging, and diseases** (Vol. 361, No. 1, 2015)	B. Fritzsch, M. Knipper, E. Friauf	K. Unsicker	25	399
**Reproductive systems biology tackles global issues** (Vol. 363, No. 1, 2016)	P. Sutovsky, A.S. Cupp, W. Thompson, M. Baker	K. Unsicker	24	312
**Wound healing and fibrosis – two sides of the same coin** (Vol. 365, No. 3, 2016)	D. Gullberg, D. Kletsas, T. Pihlajaniemi	T. Pihlajaniemi	19	241
**Recent advances in mitochondrial biology—integrated aspects** (Vol. 367, No. 1, 2017)	C. Meisinger, C. Hunte	K. Unsicker	13	159
**Development, remodeling and regeneration of the lung** (Vol. 367, No. 3, 2017)	C. Muehlfeld, M. Ochs, B. Singh	B. Singh	25	362
**Genetic kidney diseases** (Vol. 369, No. 1, 2017)	T. Huber, H. Holthofer	K. Unsicker	21	244
**Neural stem cells: developmental mechanisms and disease modeling** (Vol. 371, No. 1, 2018)	X. Zhao, D. Moore	X. Zhao	17	212
**Neutrophil biology** (Vol. 371, No. 3, 2018)	S. Liao, C. Jenne, B. Singh	B. Singh	23	253
**The sympathetic nervous system: malignancy, disease, and novel functions** (Vol. 372, No. 2, 2018)	K. Huber, I. Janoueix-Lerosey, W. Kummer, H. Rohrer, A.S. Tischler	K. Unsicker	23	280
**Parkinson’s disease: Molecules, cells, and circuitries** (Vol. 373, No. 1, 2018)	H. Braak, K. Del Tredici-Braak, T. Gasser	K. Unsicker	24	336
**Recent advances in hippocampal structure and function** (Vol. 373, No. 3, 2018)	O. von Bohlen und Halbach, A. Draguhn, J. Storm-Mathisen	K. Unsicker	13	220
**Towards new frontiers in neuroendocrinology: a tribute to Peter H. Seeburg** (Vol. 375, No. 1, 2019)	V. Grinevich, G. F. Jirikowski	K. Unsicker	27	327
**Depression and antidepressant action—from molecules to networks** (Vol. 377, No. 1, 2019)	T. Rantamäki, I. Yalcin	K. Unsicker	9	124
**Structure, development and evolution of the digestive system** (Vol. 377, No. 3, 2019)	V. Hartenstein, P. Martinez-Serra	V. Hartenstein	15	258
**Tribute to Werner W. Franke** (Vol. 379, No. 1, 2020)	K. Unsicker	K. Unsicker	19	222
**Animal models** (Vol. 380, No. 2, 2020)	D. Meyerholz, A.P. Beck, B. Singh	B. Singh	12	209
**Cell biology of neurotrophic factors** (Vol. 382, No. 1, 2020)	M. Saarma, W. Mobley, V. Leßmann	K. Unsicker	15	200
**Olfactory coding and circuitries** (Vol. 383, No. 1, 2021)	S. Sachse, I. Manzini	K. Unsicker	40	595
**Immune-mediated kidney diseases** (Vol. 385, No. 2, 2021)	U. Panzer, T. B. Huber	K. Unsicker	16	223

## Volker Hartenstein

From its founding in 1924, the stated objective of the journal has been to draw the connection between medically relevant histology and basic cell biology. To attain this goal, a strong emphasis has always been placed on visualization of cellular and subcellular structures and on the technologies, including electron microscopy, histochemistry, immunohistochemistry, and all its modern derivatives required for visualization.

Throughout the long time he has been holding the leadership of the journal, Professor Klaus Unsicker has striven to stay true to this goal. He helped to divide the task of screening, reviewing, and editing submitted articles among a team of section editors who specialized along the lines of tissue and organ systems, and this way of organizing the division of labor has proven very successful, in particular also in view of devising the large number and broad scope of special issues that have been published over the past three decades. I had the gratifying task to act as section editor for invertebrate systems since just before the turn of the century, and I look back with satisfaction and happiness to the many positive interactions I had with Klaus over these years, in discussing scientific aspects of articles or special issues, matters of how to improve the journal, or simply events of our own professional and private lives. Without a doubt, the in-person editorial meetings that Klaus put great effort into organizing were a great help in fostering this kind of interaction.

I considered my role as section editor for invertebrate systems mainly in identifying scientific contributions that investigate novel aspects of cell structure, function, or development in various invertebrate systems, while at the same time emphasizing the relevance of these data to mammalian tissue organization and pathology. The close connections in molecular and cellular structure that exist between all animals have become abundantly clear through the long series of discoveries in the fields of developmental biology and molecular genetics of the last 40 years, a fact that underlines the importance of a comparative outlook, integrating new cell biological findings in a particular species into a wider evolutionary context. Long before the developmental-genetic nexus that unites all animals had become apparent, the journal was one of the leading forums where ultrastructural and experimental studies on a great variety of cell types were published, studies which would serve as classical works of reference up to the present day. To name but one representative topic, the digestive epithelium, which I had the opportunity to cover in a recent special issue (Structure, Development and Evolution of the Digestive System, Vol. 377, No. 3, 2019) in close collaboration with Klaus: close to a dozen of the original descriptions of the zymogenic, absorptive, and phagocytic functions of enterocytes in virtually all animal phyla were published in CTR. Klaus, while realizing that to fulfill the mission of CTR in bringing together basic cell biology and pathology it was important to put the main emphasis of the journal on research in vertebrate tissues, also strongly supported the comparative evolutionary and developmental approach, not least in his prolific activity to put together special issues that revolved around this approach, special issues (to name but a few) like “Molecular bases of limb and muscle development” (Vol. 296, No. 1, 1999), “Recent advances in developmental neuroscience” (Vol.305, No.2, 2001), “The circadian system: circuits-cells-clock genes” (Vol. 309, No. 1, 2002), “Cell-to cell communication: current views and future perspectives” (Vol. 352, No.1, 2013), or “Structure and evolution of the digestive system” (Vol. 377, No. 3, 2019). The journal CTR owes Klaus a great Thank you for its continued success and congratulates him to his 80th birthday.

## Horst-Werner Korf

During my long-lasting service as section editor of “Neurondocrinology,” Professor Klaus Unsicker has continuously provided encouragement, support, and stimulating advice. In particular, he has always followed the development of circadian research with great interest and his suggestion to compose a special issue on this topic has been one of my most exciting experience as section editor. This special issue (Vol.309, No.1, 2002) has provided a comprehensive survey of the system, and several articles have reached the list of most cited papers of Cell and Tissue Research, those on the suprachiasmatic nucleus by Moore et al. (2002) and on melatonin receptors by von Gall et al. (2002) being in the top of the list. On behalf of the neuroendocrine and circadian community and personally, I wish Klaus Unsicker all the best for the next decade and his future endeavors.

## Xinyu Zhao

With fast development of the stem cell field in early 2000 came a significant increase in stem cell manuscripts submitted to CTR. I was recruited by Klaus to serve as the section editor of stem cells for the journal in 2014. It was my first time to serve as an editor for a scientific journal, and the guidance from Klaus and others were critical during my first few years. With the encouragement from Klaus, I started planning for a special issue on neural stem cells (NSCs) in 2016. NSCs are multipotent stem cells that can self-renew and have the capacity to differentiate into other cell types in the central nervous systems. NSCs exist naturally in embryonic development and adult brains and therefore have the exciting potential for endogenous neural repair. On the other hand, NSCs can also be differentiated from pluripotent embryonic stem cells (ESCs) and induced pluripotent stem cells (iPSCs). The fast advancement of human iPSC field leads to escalating interest in NSCs that has not slowed down even now, because NSCs provide not only models for studying mechanisms of human brain development but also experimental systems for understanding pathogenesis of human diseases. A special issue on NSCs would be a timely contribution to the field. Darcie Moore who was a new assistant professor at University of Wisconsin-Madison working on stem cell aging graciously agreed to serve as the co-editor with me for the NSC special tissue. After 1 year of planning and writing, we finally published the special issue “Neural stem cells: developmental mechanisms and disease modeling” in January 2018 (Vol. 371, No. 1). The special issue contains one editorial written by Darcie and myself and 17 reviews articles covering NSCs in development, adult, and differentiated from ESCs and iPSCs. The special issue has been well received with 17 articles being cited multiple times, 14 out of 18 articles being cited at least 10 times, and the article on lncRNA (Andersen and Lim 2018) being cited 46 times. Klaus’s vision and leadership were instrumental in the success of the special issue as well as the stem cell aspects of CTR.

## Lihong Shi

It has been 2 years since I became an editor for CTR. This is the first time for me to serve as an editor for a scientific journal and I am so excited. During this period, I have received great help from Klaus and others. I am deeply impressed by Klaus’ enthusiasm to science and his responsibility to make CTR a better journal. For me, the editorial board of CTR feels like a family. I wish CTR becomes even greater in the future under Klaus’ leadership.

## David Kelsell

Twelve years or so ago, Klaus contacted me to enquire if I was interested in overseeing the development of a new section that reflected the impact of developing understanding of the genetic and molecular mechanisms underlying both inherited and acquired diseases. I was delighted to be involved, and we decided to call the section Disease Mechanisms. It became a Cell and Tissue Research subject area in October 2009 with the scope of inviting reviews and papers that combine molecular genetics and pathology towards understanding the pathways and cellular changes in human disorders. I have been on the CTR board over 12 years and have really enjoyed our board meetings and dinners together. One year we all ended up watching the World Cup final and Germany won! The scenes afterwards in the streets of Heidelberg were joyful and noisy! I was invited to the board by Klaus after a recommendation by fellow board member Dr. Werner Franke as we have a mutual interest in cell junctions, in particular the desmosomal protein Desmoplakin. During my time on the board, I have overseen two special issues. One, of course, was on the topic of “Junctions in human health and inherited disease” and the other on “Current insights into protease dynamics in human epithelial disease and barrier function.” I thank the time and enthusiasm that my co-leads injected into these special editions: Dr. Spiro Getsios, Prof. Andy Forge, and Prof. Mike Curtis and the authors of all the articles plus, of course, the support and encouragement from Klaus as the publication deadlines approached! It is a pleasure working with Klaus on CTR; he brings enthusiasm, reality, and humor to our board meetings. He should be very proud of his editorial leadership and vision he brings to CTR particularly as articles and reviews span such a diverse range of topics and indeed species.

## Taina Pihlajaniemi

Working in the editorial board of CTR as section editor for the topic of Extracellular Matrix has been a great privilege on account of the high standards of the journal itself and, especially, because of its unsurpassable Editor-in-Chief, Klaus Unsicker. His dedication to the journal and to supporting the section editors is exemplary. The applauds should also be extended to the editorial assistance staff, which works in close unity with the editor-in-chief in getting reviews and each journal issue compiled in time. I consider Klaus as a champion and hero for advancing science both through his understanding of biological research as well as the ins-and-outs of journal publishing. It is not often that one can congratulate a colleague that is still actively pursuing his academic tasks at the age of Klaus. Dear Klaus, my warmest thanks for the wonderful guidance and inspiration that you have constantly provided, congratulations for your high achievements as a scientist and an editor, and best wishes for the years to come.

## Peter Sutovsky

Reproductive Biology Section was formally established in 1999 by recruiting Dr. David De Kretser as section editor, followed by Dr. Gail Risbridger helming the section from 2002 through 2006. Since 2007, I have served as section editor. The high volume of reproductive biology themed manuscripts prompted in 2021 the recruitment of Dr. Stephanie Pangas as the section editor for Female Reproductive Biology, while I stayed on board as section editor for Male Reproductive Biology. Introduction of monothematic special issues overseen by individual section editors was one of many innovations implemented during Professor Unsicker’s tenure. Since inception of the reproductive section, three special issues on reproduction have been published, with mostly review articles focusing on select topics in reproductive biology. The first special issue on reproductive biology published as Vol. 322, No. 1, in October 2005, with introduction by Professor Unsicker (2005) set the theme of “reproduction, development and the early origins of adult disease.” This special issue contains the most frequently cited reproductive biology-oriented article on CTR record (Lewis and Aitken 2005). This review article discussed not only the impact of sperm DNA damage on human fertility, focusing on fertilization, pregnancy establishment, and spontaneous pregnancy loos, but also the implications of paternal genome integrity for health and disease in offspring and transgenerational health impacts. Other highly cited articles discussed the response of female reproductive system to male seminal plasma factors (Robertson 2005) and the regulation of male reproductive function by stress hormones (Hardy et al. 2005).

The second reproductive biology special issue (Vol. 349, No. 3, 2012) was subtitled “Cell Biology Solves the Mysteries of Reproduction” and aimed to showcase the state of the art of research summarized in 17 review articles and one original research paper. Topics included gametogenesis and gonadal function, fertilization, stem cells, microRNAs, pregnancy establishment, endometriosis, proteomic applications for fertility testing, mitochondrial inheritance and disease, reproductive effects of diabetes, and epigenetics of adult disease. The most frequently cited paper was the review of gene expression patterns in the mammalian epididymis by Belleannée et al. (2012).

The theme of the third special issue on reproductive biology (Vol. 363, No. 1, 2016) was reproductive systems biology tackling global issues, including population growth, food safety, and reproductive health. Reflecting on the importance of reproductive biology for health, medicine, and food animal agriculture, the issue included 23 review articles covering ovarian and testicular function, gametogenesis, fertilization, preimplantation embryo development, pregnancy establishment, omics, and bioengineering, covering studies in humans, biomedical animal models, and agriculturally important livestock species that are now making inroads into biomedical research as large animal models. Among the most frequently cited articles in this issue, and in CTR in general, is the review of gamete transport and sperm interactions with female reproductive tract by Susan Suarez (2016).

By publishing in CTR under the leadership of Professor Unsicker, reproductive biologists have enjoyed unique opportunities to reach and appeal to general readership beyond the immediate focus areas of human and animal reproduction, fertility, and development, also including comparative studies on wildlife species and seldom used biomedical animal models.

## Baljit Singh

As many would have done already, I must confess it was a shocker to learn that Klaus is 80 years old! He looks far younger than that. It has been a privilege and an honor to work with Klaus for nearly 15 years in my role as section editor for Immunology and Inflammation in CTR. There was lots of learning from Klaus during this period. He always demonstrated an unwavering commitment and focus on the quality of the manuscripts accepted for publication in CTR and the production quality of the print journal. There were a couple of occasions when he alerted me to the issues with the quality of the figures in manuscripts accepted by me. The memories can play tricks on us, but if I recall correctly, there were times when Klaus clearly expressed his frustration on the production quality of the print issues of CTR and may be even asked for reprinting the whole issue! Klaus leads by example as shown by his rigor and hard work in bringing to life nearly 65% of the CTR’s 46 special issues. Just to put it in perspective, my own contribution of four special issues (Innate Immunity, Development, Remodelling and Regeneration of the Lung; Neutrophil Biology; Animal Models) pales in comparison. Similar to my other colleagues on the editorial board, I always looked forward to the annual meeting of the editorial board to review the progress and to plan new tasks. The memories of the one such meeting when we all watched the FIFA World Cup final together, yes, the one in which Germany won accompanied by a deafening roar from the streets of Heidelberg will remain fresh in my mind for a long time. Therefore, with all of his youthfulness, I am left wondering if he is really 80 years old or is it an excuse to just slow down! Thank you, Klaus, for the opportunity and privilege to work with you to serve the CTR, our nearly 100-year-old journal!

Together with many colleagues all over the world, we thank Klaus Unsicker for his constructive advice and valuable support. Ad multos annos!

On behalf of the Editorial Board.

Volker Hartenstein, David Kelsell, Horst-Werner Korf, Taina Pihlajaniemi, Lihong Shi, Baljit Singh, Peter Sutovsky, Xinyu Zhao.

## References

[CR1] Andersen RE, Lim DA (2018). Forging our understanding of lncRNAs in the brain. Cell Tissue Res.

[CR2] Belleannée C, Thimon V, Sullivan R (2012). Region-specific gene expression in the epididymis. Cell Tissue Res.

[CR3] Franke WW (2012). Klaus Unsicker—still going strong: congratulations on his seventieth birthday. Cell Tissue Res.

[CR4] Hardy MP, Gao HB, Dong Q, Ge R, Wang Q, WR, Feng X and Sottas C,  (2005). Stress hormone and male reproductive function. Cell Tissue Res.

[CR5] Korf HW (2012). Klaus Unsicker: in honor of his seventieth birthday. Cell Tissue Res.

[CR6] Lewis SEM, Aitken RJ (2005). DNA damage to spermatozoa has impacts on fertilization and pregnancy. Cell Tissue Res.

[CR7] Moore RY, Speh JC, Leak RK (2002). Suprachiasmatic nucleus organization. Cell Tissue Res.

[CR8] Robertson SA (2005). Seminal plasma and male factor signalling in the female reproductive tract. Cell Tissue Res.

[CR9] Suarez SS (2016). Mammalian sperm interactions with the female reproductive tract. Cell Tissue Res.

[CR10] Unsicker K (2005). Reproduction, development, and the early origins of adult disease. Cell Tissue Res.

[CR11] von Gall C, Stehle JH, Weaver DR (2002). Mammalian melatonin receptors: molecular biology and signal transduction. Cell Tissue Res.

